# Genetic elements associated with antimicrobial resistance in enteropathogenic *Escherichia coli *(EPEC) from Brazil

**DOI:** 10.1186/1471-2180-10-25

**Published:** 2010-01-27

**Authors:** Isabel CA Scaletsky, Tamara B Souza, Katia RS Aranda, Iruka N Okeke

**Affiliations:** 1 Departamento de Microbiologia, Immunologia e Parasitologia, Universidade Federal de São Paulo, Rua Botucatu, 862, São Paulo, 04023-062, Brazil; 2 Departamento de Pediatria, Universidade Federal de São Paulo, Rua Botucatu 598, São Paulo, 04023-062, Brazil; 3 Department of Biology, Haverford College, 370 Lancaster Avenue, Haverford PA, 19041, USA

## Abstract

**Background:**

We recently observed an association of resistance with a certain enteropathogenic *Escherichia coli *(EPEC) serotypes and identified a conjugative plasmid, similar to plasmid pED208, that was conserved among archival O111:H2/NM and O119:H2 strains of diverse geographical origin. In this study, we sought to determine the prevalence and distribution of this plasmid among a collection of EPEC isolates from Brazil, as well as to study the susceptibilities of these isolates to antimicrobial agents.

**Results:**

Resistance was more commonly seen in typical EPEC than atypical strains. The most prevalent resistances were to ampicillin, tetracycline, streptomycin and the sulfonamides. Markers for the EPEC conjugative multiresistance plasmid, were detected in 21 (30%) of typical but only 4 (5%) of atypical strains (p = 0.001, Chi-squared test). This plasmid, previously reported from only O111 and O119 strains was found in O55 and O127 strains and was associated with the presence of class 1 integrons.

**Conclusion:**

Our data suggest a limited but expanding host range for the EPEC resistance plasmid.

## Background

Enteropathogenic *Escherichia coli *(EPEC) is an important cause of infantile diarrhea worldwide and particularly in developing countries [1,2]. EPEC strains adhere intimately to the brush border of the intestinal epithelium and initiate a complex signaling cascade by virtue of a chromosomal pathogenicity island, the locus for enterocyte effacement (LEE) (reviewed by Clarke et al [3]). EPEC strains also carry an EPEC adherence factor (EAF) plasmid, which encodes the bundle forming pili, a plasmid-encoded regulator, and other putative virulence genes. The majority of EPEC isolates belong to classic serotypes derived from 12 classical O serogroups (O26, O55, O86, O111, O114, O119, O125, O126, O127, O128, O142, and O158) [4,5]. A robust genetic definition, and molecular and cellular detection methods, have made it possible to expand the repertoire of known EPEC lineages, identifying other EPEC that possess the LEE but do not harbor the EAF plasmid [5,6]. Thus, EPEC strains harboring the EAF plasmid are classified as "typical EPEC", while strains which do not harbor the EAF plasmid, are classified as atypical EPEC" [7].

For many decades, typical EPEC was the main bacterial enteropathogen in infants in Brazil. Several studies in the 1980s and early 1990s showed a high frequency of typical serotypes, particularly serotypes O111:H2 and O119:H6 [2,8-16]. However, some recent studies have shown a decrease in the isolation rates of these serotypes accompanied by and an apparent increase in the frequency of atypical EPEC [9,10,17-20]. Most atypical EPEC strains belong to traditional EPEC serogroups, but several strains of non-EPEC serogroups have also been identified in different epidemiological studies [9,10,17,21].

Although most EPEC infections resolve without antimicrobial therapy, antimicrobials should be administered in persistent infections, where the choice of effective antimicrobials may be crucial for patient recovery and even survival [22]. In addition to a selective pressure, specifically directed towards EPEC, the persistence of resistant EPEC strains is even more likely to be related to selective pressure from antimicrobials applied at the population level. There is considerable evidence to suggest that young children, those most vulnerable to EPEC infection, are at risk of infection with resistant commensals, as well as pathogens, from their caregivers and household contents [23,24]. Therefore resistance genes acquired and recombined in other niches may present in EPEC strains from infants.

Many isolated enteric bacterial are known to harbor mobile elements that encode antimicrobial resistance. For example, apparently successful conserved elements have recently been described in *Salmonella *serovars and *Yersiniae *[25,26]. We recently observed an association of resistance with a certain EPEC serotypes and identified a conjugative plasmid, similar to plasmid pED208, that was conserved among archival O111:H2/NM and O119:H2 strains of diverse geographical origin [27]. However the distribution and therefore significance of this element is yet to be studied more broadly, particularly in recent isolates. In this study, we sought to determine the prevalence and distribution of this plasmid among a collection of EPEC isolates from Brazil, as well as to study the susceptibilities of these isolates to antimicrobial agents.

## Results and Discussion

We assessed resistance in 149 EPEC strains (70 typical and 79 atypical) isolated from Brazilian children in previously described studies [9,10,21]. Typical EPEC isolates were commonly resistant to ampicillin, tetracycline, streptomycin and the sulfonamides (Table 1). All but 14 (20%) typical strains were resistant to at least one antimicrobial and 30 (43%) of the strains were resistant to three or more of the antimicrobials tested. Atypical EPEC strains were much less likely to be resistant to ampicillin, tetracycline, streptomycin and the sulfonamides, but were more likely to show resistance to trimethoprim. Although resistance to quinolones and extended-spectrum beta-lactams has emerged among enteric organisms, all the strains tested in this study were susceptible to these drugs.

**Table 1:** Antimicrobial resistance of EPEC isolates from Brazil

Antimicrobial	N° (%) of resistant EPEC isolates:	
	
	tEPEC (*n *= 70)	aEPEC (*n *= 79)
Ampicillin	42 (60)	19 (24)
Chloramphenicol	14 (20)	2 (2.5)
Kanamycin	0	0
Sulphonamide	44 (62.8)	20 (25.3)
Streptomycin	24 (34.3)	8 (10.1)
Tetracycline	30 (42.8)	8 (10.1)
Trimethoprim	1 (1.4)	13 (16.4)
Ceftazidime	0	0
Ciprofloxacin	0	0
Lomefloxacin	0	0
Ofloxacin	0	0
Nalidixic acid	0	0

EPEC strains bearing the recently reported resistance plasmid, which we sought in this study, carry at least two, and sometimes more than three, large plasmids [27]. Additionally, because the plasmid is only partially conserved, plasmid profiling cannot be used to study its distribution. Instead, we used primers that recognize *traI *and *traC *genes from the conjugative transfer region of this resistance plasmid, and the closely related plasmid pED208, to screen the recent Brazilian EPEC isolates for the presence of this element by PCR [27]. We have previously demonstrated that these primers do not produce amplicons with other known conjugative plasmids, other than those related to pED208 [27]. We additionally screened the strains for a *trbC-traU *region that is present in pED208 but absent from the EPEC multiresistant plasmid. All the strains screened in this study failed to produce an amplicon with this primer pair.

As shown in Table 2, both the *traI *and the *traC *amplicons were produced in 21 (30%) of typical but only 4 (5%) of atypical strains (p = 0.001, Chi-squared test). Moreover, 18 (26%) typical EPEC but only 5 (6%) atypical EPEC produced an amplicon with at least one of the primers pairs (p = 0.001). Of the 9 atypical EPEC that possessed the *traI *and/or *traC *marker, four belonged to O55 or O119 serogroups, which are associated with typical EPEC (see Additional file 1). These strains were negative for EAF and *bfpA *probes, but they were positive for *perA*, an EAF gene [21]. Therefore, like some other atypical strains that have been described in the literature [28-30], these strains carry vestiges of the EAF plasmid.

**Table 2:** Occurrence of EPEC conjugative multiresistance plasmid loci and plasmid replicons among EPEC isolates from Brazil

**Gene or Replicon**	**No. (%) of isolates positive:**	
	
	**tEPEC** **(*n *= 70)**	**aEPEC** **(*n *= 79)**
**Conjugative genes**		
*traI*	11 (15.7)	3 (3.8)
*traC*	7 (10)	2 (2.5)
*traI*+*traC*	21 (30)	4 (5.1)
		
**Class 1 integrons**		
*aadA1*	12 (17.1)	1 (1.3)
*sulII*	25 (35.7)	3 (3.8)
*tetA*	14 (20)	0
*Cat*	13 (18.6)	1 (1.3)
*merA*	3 (4.3)	0
		
**Replicons**		
B/O	1 (1.4)	1 (1.3)
FIC	0	1 (1.3)
A/C	1 (1.4)	3 (3.8)
P	1 (1.4)	0
T	0	0
K/B	11 (15.7)	15 (18.9)
W	3 (4.3)	21 (26.6)
FHA	0	0
FIA	0	0
FIB	32 (45.7)	23 (29.1)
Y	0	1 (1.3)
I1	11 (15.7)	3 (3.8)
Frep	1 (1.4)	17 (21.5)
X	0	0
HI1	0	0
N	0	0
HI2	0	0
L/M	0	0

Our data show that the EPEC resistance plasmid is found commonly in typical EPEC, and is uncommon in atypical EPEC, consistent with earlier data [27]. However, previous evaluation of the distribution of the EPEC multiresistance plasmid in a small collection of archival strains suggested that it was limited to O111:H2 and O119:H2 strains, which carry the EAF plasmid or vestiges of it [27]. In the current study, *traI *and *traC *markers from the resistance plasmid were identified in strains belonging to the serotypes O55:H6, O127:H6, and O119:H6, as well as O55 and O119 atypical strains that carry vestiges of the EAF plasmid (see Additional file 1). To determine whether this broader distribution among Brazilian isolates was a recent development, we screened 36 archival EPEC strains that were isolated in the 1970s and 1980s from children with diarrhea in São Paulo [12], and the plasmid was predominately found in O111:H2, O119:H6 and O142:H6 strains (data not shown), which were among the most common circulating serovars at that time [2,13,31].

Although isolates that were susceptible to all tested agents were more likely to be *traI *and *traC *negative and strains that had these markers were to a higher degree multiple resistant, in contrast to the association seen with older isolates from other geographic locations [27], we did not find that the presence of *traI *and *traC *markers in the EPEC isolates were absolutely or significantly associated with multiple resistance. The EPEC resistance plasmids of previously studied O111 and O119 strains bear class 1 integrons as well as one or more resistance genes identical to those on *Salmonella enterica *subsp. Typhi multiresistant plasmid pHCM1 [25]. Some typical strains and all atypical strains had fewer of these markers, even though antimicrobial resistance was just as common in these isolates (see Additional file 1). Among isolates 12 of 39 strains carrying the EPEC resistance plasmid and one of 31 strains without it had a class 1 integron (p = 0.0025, Yates corrected Chi-squared test). None of the other markers screened showed significant association with the plasmid in strains. Combined with the resistance data, these findings suggest that the EPEC resistance plasmid plays less of a role in conferring resistance in these EPEC isolates, in particular atypical strains, and that there may be possible other genetic elements conferring resistance among those strains.

EPEC strains bearing the EPEC resistance plasmid carry at least two, and sometimes more than three, large plasmids [27,30]. We used a PCR-based replicon typing scheme to determine other possible plasmid types conferring antimicrobial resistance in the EPEC strains studied. Our results indicate that both typical and atypical strains harbor plasmid types which have been shown to carry mobile genetic elements encoding drug-resistance. In addition, significant differences in plasmid replicon content were observed between typical and atypical EPEC strains (Table 2). In particularly, the IncI1 replicon occurred significantly more often among typical strains, whereas the IncFrep replicon was observed significantly more often among atypical strains (p = 0.013 and p = 0.001, respectively) The IncT, IncFIIA, IncFIA, IncX, IncHI1, IncN, IncHI2, and IncL/M replicons were not detected in any of the strains. Among the replicon profiles identified, IncFIB occurring alone was the most common (see Additional file 1).

Antimicrobial resistance is increasing worldwide. Resistance in intestinal organisms is of interest it can compromise treatment of infections caused by pathogenic strains but also because the gut is a complex, diverse and heavily populated niche and resistant organisms there can transmit resistance genes horizontally. Many investigators have documented a high prevalence of antimicrobial resistance among EPEC strains in different parts of the world but few of these studies have been performed on recent isolates [22,32-35]. Resistance appeared at the beginning of the antibiotic era and epidemiological data suggests that its prevalence is associated with the 1970s and 1980s and diversity of antimicrobial use [33,35]. The genetic basis for this resistance and the evolutionary consequences are rarely studied.

## Conclusion

Our data show that the EPEC resistance plasmid is found commonly in typical EPEC, and is uncommon in atypical EPEC, consistent with earlier data. However, previous evaluation of the distribution of the EPEC multiresistance plasmid in a small collection of archival strains suggested that it was limited to O111:H2 and O119:H2 strains, which carry the EAF plasmid or vestiges of it. In this study, the host range of the EPEC resistance plasmid, although still largely restricted to typical EPEC, was seen to be greater in recent isolates.

## Methods

### Bacterial strains

The 149 strains examined in this report were isolated between 1997-1999 during an epidemiological study of acute diarrhea in children <2 years of age conducted in different regions of Brazil and between 2002 to 2003 from children <5 years of age with diarrhea in São Paulo [9,10,21]. These strains were identified by hybridization with *eae *and/or EAF probe sequences and serotyped. Most of these EPEC strains had also been characterized by the presence of LEE-associated DNA sequences, and *bfpA *and *perA *sequences, and adherence to HEp-2 cells [21].

### Preparation of bacterial DNA and PCR amplification for detection of the EPEC conjugative multiresistance plasmid, class 1 integron and plasmidreplicons

The bacterial DNA was extracted from a single colony on a LB agar plate. The bacteria were suspended in 500 μl of 1X phosphate-buffered saline (pH 7.4) solution, boiled for 10 min, and centrifuged. Two microliters of the resulting supernatant was used as template DNA in 25 μl of the PCR mixture. Each reaction mixture contained 0.4 mM deoxynucleoside triphosphates, 1 U of Taq polymerase, 1 × Taq reaction buffer, and 2 μM of each of the primers. Primer sequences and PCR conditions used were as previously published [27]. Strains were screened for the presence of the plasmid pMB80 by PCR using primers complementary to internal regions of the *traI *and *traC *genes that are conserved between the MB80 *tra *system and the closely related plasmid pED208, as well as one primer pair whose product straddles *traU *and *trbC *in pED280 in a region not conserved in pMB80 [27]. Strains were screened for class 1 integrons using primers designed by Levesque et al[36] as well as for the presence of *sulII *gene (conferring sulphonamide resistance), *tetA *gene (tetracycline resistance), trimetroprim resistance gene, *cat *(kanamycin resistance), *strAB *(streptomycin resistance), and a *mer *operon (mercury resistance) using primers originally designed for the *Salmonella *enteric serovar Typhi multiresistant plasmid, pHCM1 [25]. EPEC strains were examined for the presence of 18 plasmid replicons using three multiplex panels described by Johnson et al. [37]. PCR products were visualized on a 1.5% agarose gel stained with ethidium bromide and visualized under UV transillumination.

### Antimicrobial susceptibility test

All strains were tested for their susceptibility to 12 antimicrobial agents commonly used in Brazil [38,39] by the broth microdilution method according to the Clinical Laboratory Standards Institute [40]. Minimal inhibition concentration (MIC) breakpoint levels and concentration of each antimicrobial were based on those specified by the CLSI. Intermediately susceptible strains were recorded as being susceptible. *E. coli *strain 25922 (ATCC) was used as the reference strain. All strains were examined for resistance to ampicillin, ceftazidime, ciprofloxacin, chloramphenicol, kanamycin, lomefloxacin, ofloxacin, streptomycin, nalidixic acid, sulfonamide, tetracycline, and trimethropin.

## Authors' contributions

ICAS and INO conceived the study and wrote the paper. TBS and KRSA performed the laboratory studies. All authors read and approved the final manuscript.

## Supplementary Material

Additional file 1 **Resistance phenotypes, markers for the EPEC conjugative multiresistance plasmid and plamid replicons in EPEC isolates**. Antimicrobial resistance phenotypes, markers for the EPEC conjugative multiresistance plasmid loci and plasmid replicon types in 149 EPEC (70 typical and 79 atypical) strains isolated from Brazil. Click here for file
