# Supplementary material for: Genetic elements associated with antimicrobial resistance in enteropathogenic Escherichia coli (EPEC) from Brazil
Source: BMC Microbiol. 2010 Jan 27;10:25. doi: 10.1186/1471-2180-10-25 (PMC2828443; doi:10.1186/1471-2180-10-25)
Supplement: Additional file 1 — Resistance phenotypes, markers for the EPEC conjugative multiresistance plasmid and plamid replicons in EPEC isolates. Antimicrobial resistance phenotypes, markers for the EPEC conjugative multiresistance plasmid loci and plasmid replicon types in 149 EPEC (70 typical and 79 atypical) strains isolated from Brazil. [file 1471-2180-10-25-S1.DOC]

**Additional file 1.** **Resistance phenotypes, markers for** **the EPEC conjugative multiresistance plasmid and plamid replicons in EPEC isolates**

| Serotype  (no. of strains) | Resistance phenotype  (no. of strains) | Conjugative genes | | | Class I  integron | ClassI integron cassette | | | | Replicon  type |
| --- | --- | --- | --- | --- | --- | --- | --- | --- | --- | --- |
|  |  | *traI* | *traC* | *traI/traC* |  | *sulII* | *tetA* | *cat* | *merA* |  |
| **Typical EPEC** |  |  |  |  |  |  |  |  |  |  |
| O2:H2;H45 (2) | AMP-CHL-STR-TET-SUL (1) | - | - | +(1) | +(1) | +(1) | - | +(1) | +(1) | K/B +FIB |
| O55:NM;H6 (15) | AMP-STR-TET-SUL (7) | +(1) | +(1) | +(7) | +(2) | +(7) | +(7) | +(4) | +(2) | variable |
|  | AMP-CHL-SUL (3) | - | - | +(2) | - | +(1) | - | - | - | variable |
| O86:H34 (2) | AMP-STR-SUL (2) | +(2) | - | - | +(1) | +(1) | - | - | - | - |
| O101:H33 (1) | AMP-CHL-STR-TET-TMP-SUL (1) | - | - | - | - | - | - | - | - | P+W+FIB+I1 |
| O111:NM;H2 (9) | AMP-STR-TET-SUL (2) | +(2) | +(1) | - | +(1) | +(3) | +(1) | - | - | FIB |
|  | STR-SUL (2) | - | - | - | - | - | - | - | - | K/B+FIB |
| O119:NM;H6 (25) | AMP-TET-SUL (12) | +(5) | +(5) | +(6) | +(7) | +(7) | +(3) | +(8) | - | variable |
|  | AMP-CHL-SUL (5) | - | - | - | - | - | - | - | - | variable |
| O127:NM;H6 (4) | AMP-STR-TET-SUL (3) | - | - | +(3) | - | +(3) | +(3) | - | - | variable |
| O145:HNT (1) | - | +(1) | - | - | - | - | - | - | - | FIB |
| O157:HNT (2) | - | - | - | - | - | - | - | - | - | FIB |
| O162:NM;H33 (2) | AMP-CHL-STR-TET-SUL (2) | - | - | - | - | - | - | - | - | FIB |
| ONT:H45;HNT (7) | AMP-STR-SUL (2) | - | - | +(1) | - | +(2) | - | - | - | FIB+K/B+W |
|  | AMP-CHL-STR-TET-SUL (2) | - | - | +(1) | - | - | - | - | - | FIB |
| Total | | 11 | 7 | 21 | 12 | 25 | 14 | 13 | 3 |  |
| **Atypical EPEC** |  |  |  |  |  |  |  |  |  |  |
| O26:NM (13) | AMP-TMP-SUL (2) | - | - | - | - | - | - | - | - | W+FIB |
|  | AMP-TET-TMP-SUL (1) | - | - | - | - | - | - | - | - | FIB |
| O33:H6 (1) | - | - | - | - | - | - | - | - | - | FIB |
| O35:H19 (1) | - | - | - | - | - | - | - | - | - | - |
| O55:NM (5) | AMP-STR-TET-SUL (1) | - | - | - | - | - | - | - | - | W |
|  | AMP-STR-SUL (1) | +(1) | - | +(1) | - | +(1) | - | - | - | A/C +Frep |
| O85:H40 (1) | TMP (1) | - | - | - | - | - | - | - | - | B/O+W |
| O101:NM (1) | AMP-TET-SUL (1) | - | - | - | - | - | - | - | - | W |
| O103:NM (2) | AMP-TMP-SUL (2) | - | - | - | - | - | - | - | - | W+FIB |
| O105:H7 (1) | - | - | - | - | - | - | - | - | - | K/B+FIB+W |
| O108:H31 (1) | - | - | - | - | - | - | - | - | - | W+Y |
| O109:H54 (1) | - | - | - | - | - | - | - | - | - | - |
| O111:NM (5) | TET-TMP-SUL (2) | - | - | - | - | - | - | - | - | K/B+W |
| O114:NM (1) | - | - | - | - | - | - | - | - | - | K/B+W+Frep |
| O119:NM;H2 (11) | TET-TMP-SUL (1) | - | - | - | - | - | - | - | - | K/B+FIB |
|  | AMP-CHL (1) | +(1) | - | +(1) | - | - | - | - | - | W+FIB |
| O126:NM (1) | - | - | - | - | - | - | - | - | - | Frep |
| O127:NM;H40 (4) | AMP-TET-SUL (1) | - | - | - | - | - | - | - | - | K/B+W |
| O128:HNT (3) | AMP-TMP-SUL (1) | - | - | - | - | - | - | - | - | W |
| O141:NM (1) | AMP-TET (1) | - | - | - | - | - | - | - | - | A/C+K/B+I1 |
| O142:HNT (8) | AMP-STR-TMP-SUL (2) | - | - | - | - | - | - | - | - | K/B+FIB |
|  | AMP-CHL (1) | +(1) | - | - | - | +(1) | - | - | - | Frep |
| O156:H16 (1) | - | - | - | +(1) | - | - | - | - | - | FIB |
| ONT:H18;NM (17) | AMP-STR-SUL (4) | - | +(2) | +(1) | +(1) | +(1) | - | +(1) | - | variable |
|  | TMP-SUL (1) | - | - | - | - | - | - | - | - | FIB+Frep |
| Total | | 3 | 2 | 4 | 1 | 3 | 0 | 1 | 0 |  |

Antimicrobial agents: AMP, ampicillin; CHL, chloramphenicol; KAN, kanamycin; STR, streptomycin; SUL, sulfamethoxazole;

TET, tetracycline; TMP, trimethoprim.
